# How many trials are needed in kinematic analysis of reach-to-grasp?—A study of the drinking task in persons with stroke and non-disabled controls

**DOI:** 10.1186/s12984-021-00895-3

**Published:** 2021-06-15

**Authors:** Gunilla Elmgren Frykberg, Helena Grip, Margit Alt Murphy

**Affiliations:** 1 Department of Neuroscience, Rehabilitation Medicine, Uppsala University, Box 256, 751 05 Uppsala, Sweden; 2 Department of Radiation Sciences, Biomedical Engineering, Umeå University, Umeå, Sweden; 3 Institute of Neuroscience and Physiology, Clinical Neuroscience, Rehabilitation Medicine, Sahlgrenska Academy, University of Gothenburg, Gothenburg, Sweden

**Keywords:** Kinematics, Upper extremity, Drinking task, Functional assessment, Performance stability, Test–retest reliability, Stroke, Non-disabled

## Abstract

**Background:**

Kinematic analysis of the 3D reach-to-grasp drinking task is recommended in stroke rehabilitation research. The number of trials required to reach performance stability, as an important aspect of reliability, has not been investigated for this task. Thus, the aims of this study were to determine the number of trials needed for the drinking task to reach within-session performance stability and to investigate trends in performance over a set of trials in non-disabled people and in a sample of individuals with chronic stroke. In addition, the between-sessions test–retest reliability in persons with stroke was established.

**Methods:**

The drinking task was performed at least 10 times, following a standardized protocol, in 44 non-disabled and 8 post-stroke individuals. A marker-based motion capture system registered arm and trunk movements during 5 pre-defined phases of the drinking task. Intra class correlation statistics were used to determine the number of trials needed to reach performance stability as well as to establish test–retest reliability. Systematic within-session trends over multiple trials were analyzed with a paired t-test.

**Results:**

For most of the kinematic variables 2 to 3 trials were needed to reach good performance stability in both investigated groups. More trials were needed for movement times in reaching and returning phase, movement smoothness, time to peak velocity and inter-joint-coordination. A small but significant trend of improvement in movement time over multiple trials was demonstrated in the non-disabled group, but not in the stroke group. A mean of 3 trials was sufficient to reach good to excellent test–retest reliability for most of the kinematic variables in the stroke sample.

**Conclusions:**

This is the first study that determines the number of trials needed for good performance stability (non-disabled and stroke) and test–retest reliability (stroke) for temporal, endpoint and angular metrics of the drinking task. For most kinematic variables, 3–5 trials are sufficient to reach good reliability. This knowledge can be used to guide future kinematic studies.

## Background

Analysis of multi-joint 3D kinematics is needed to understand the underlying mechanisms of the altered movement strategies commonly seen post stroke [1]. Unlike traditional clinical assessments, objective measures of movement quality allow differentiation between behavioral recovery and compensation in evaluation of treatment effects [2–4]. Here, the kinematic analysis can provide detailed and objective information about movement performance and movement quality during everyday activities, such as reach-to-grasp [5, 6].

Reach-to-grasp is frequently used in daily activities and its performance in non-disabled individuals is characterized by efficient spatiotemporal coordination of the arm and hand segments for transport and grasping [7]. Regaining arm- and hand function post-stroke is one of the highest priority goals in rehabilitation, and still about 65% of the patients with hemiparesis have impaired ability to reach, grasp and handle objects at 6 months after stroke onset [8]. Motor performance of reach-to-grasp tasks in the stroke population shows longer movement time, lower peak velocity, decreased elbow extension, greater arm abduction and trunk displacement, and decreased smoothness as compared to non-disabled controls [5, 9–11]. Among the reach-to-grasp tasks, drinking from a glass has, due to its ecological validity and ease of standardization, been recommended as a functional task for quantifying quality of movement in stroke rehabilitation research [12].

Another aspect that needs to be considered in performance of daily purposeful tasks is variability of movements. Variability is inherent in human movement control, i.e. different neuromotor processes are available to produce automatic movement strategies needed for achieving goals in daily life [13]. The concept of movement variability is defined as typical variations in motor performance when a task is repeatedly being executed [14], which is something that needs to be taken into account when conducting clinical research studies. Optimal movement variability is crucial for healthy motor control [13, 15]. A high level of automaticity and relatively constant variability is, however, expected when a well-known activity is repetitively performed [16].

Requests for standardization of kinematic analysis of upper extremity movements have been highlighted [11] and for research purposes several efforts have been made to agree on which tasks to study and which systems and metrics to use [5, 9–12]. Clinimetric properties, including reliability, validity and responsiveness, have been reported for some kinematic metrics [9, 11, 17, 18] although more studies are needed [19, 20]. One aspect of reliability that has been sparsely investigated is the performance stability of selected variables within a session of a series of trials. Most of the studies of reach-to-grasp tasks in stroke populations include 3–10 trials per task although in few studies up to 20 trials have been reported [5, 11]. A recent consensus on kinematic studies in stroke recommended at least 15 trials to be collected, both for 2D performance assays and 3D functional tasks [12].

Hence, the question of how many trials that are needed to reach performance stability of kinematic measures in goal-directed reach-to-grasp tasks remains. A previous study analyzing movement performance during fast pointing in non-disabled participants, demonstrated that 3 trials were required to reach good within-trial reliability for movement time and peak velocity, whereas up to 47 trials were required for trajectory metrics [21]. Another study in persons with subacute stroke, where also 3D motion capture was used, reported that 5 trials was sufficient to get reliable results for reaching kinematics [22].

To our knowledge, no studies have defined the number of trials needed to achieve performance stability, i.e. good reliability, in kinematic measures of goal-directed reach-to-grasp tasks, nor has this been investigated in people with disabilities. Thus, the primary aim of this study was to determine the number of trials needed to reach good performance stability of the kinematic variables during the drinking task in non-disabled people and in a sample of individuals with chronic stroke. Further, the performance stability over the set of multiple trials was investigated. In addition, the between-sessions test–retest reliability of selected kinematics in a sub-sample of individuals with stroke was established.

## Methods

### Participants

This study included 44 non-disabled participants who were recruited through personal contacts and general advertisements during 2016–2019 in the urban area of Gothenburg in Sweden. The non-disabled participants were included when they were between 30 and 85 years, had not being diagnosed with any medical condition that would potentially influence the movements of the upper extremity or upper body, and perceived themselves as healthy. Potential participants were excluded, if they showed any observable neurological signs (e.g. tremor), difficulties to follow simple instructions or had uncorrected visual acuity that influenced the movement performance. The non-disabled participants performed the kinematic drinking task at one occasion.

In addition, eight participants with stroke, screened for separate single case design studies between 2018 and 2020 were included. Inclusion criteria were a diagnosis of stroke at least 6 months earlier, ability to adhere to the upper extremity virtual reality intervention study protocol requiring ability to hold an object like remote control with the more-affected hand, and able to attend the physical visits over 15 weeks’ time at the research site [23]. For the current analysis, only data from the stable phase (phase A) prior intervention was used. Five participants with stroke had kinematic data available from four separate testing sessions (with one week apart), and three had data only from one screening session.

Background data on age, sex, hand dominance, body height and weight were registered for all participants. The type and side of stroke and time since onset were also recorded for participants with stroke. Upper extremity motor impairment in stroke was assessed with the Fugl-Meyer Assessment of Upper extremity (FMA-UE) [24, 25] and the activity limitation with the Action Research Arm Test (ARAT) [26, 27]. In addition, the non-motor domains of the FMA-UE (sensation, range of motion and pain) and muscle tone (modified Ashworth Scale) [28] for elbow and wrist joint movements were assessed. The demographic and clinical characteristics of all participants are shown in Table 1.

**Table 1:** Demographic and clinical characteristics of participants

Averaged demographic characteristics, mean (SD)		
	Non-disabled (n = 44)	Stroke (n = 8)
Age, years	59.5 (14.9)	61.2 (8.8)
Women/men	21/23	4/4
Height, cm	171.7 (9.6)	173.0 (1.4)
Weight, kg	74.6 (16.1)	73.9 (8.6)

Individual characteristics of the participants with stroke										
ID	Age	Sex	Affected arm	Stroke type	Years since stroke	FMA-UE (0–66)	ARAT (0–57)	Sens (0–12)	ROM/ pain (0–24)	mAS (0–20)
1	48	F	Right	Infarct	0.5	40	27	10	19/20	4
2	74	F	Left	Infarct	4	44	44	12	21/22	3
3	50	F	Right	Infarct	2	51	40	11	24/24	4
4	69	M	Right	Infarct	6	51	56	12	22/23	4
5	65	M	Left	Infarct	1	63	55	12	24/24	0
6	61	M	Right	Infarct	4	60	52	12	21/23	0
7	60	M	Right	Infarct	10	64	56	10	24/24	0
8	63	F	Right	Infarct	2	64	56	12	24/24	0

Participants with stroke ID 1–5 were also included in test–retest reliability analysis

*FMA-UE* Fugl-Meyer assessment of upper extremity, *ARAT* Action Research Arm test, *Sens* FMA-UE sensory impairment score, *ROM* FMA-UE range of motion, *mAS* modified Ashworth Score

The ethical approval was provided by the Swedish Ethical Review Authority (318–04, 1074–18, 1075–18), and oral and written informed consent was obtained from all participants.

### Kinematic movement analysis

The standardized established kinematic analysis testing protocol for drinking task was used [10, 12, 17]. Kinematic data was acquired with a 5-camera high speed optoelectronic motion capture system (Proreflex MCU 240 Hz, Qualisys AB, Gothenburg, Sweden). The cameras emit infra-red light that is reflected by the circular markers placed on anatomical landmarks on the body. The eight markers (12 mm) were placed on the tested hand (III metacarpophalangeal joint), wrist (styloid process of ulna), elbow (lateral epicondyle), on both shoulders (acromion), trunk (sternum), forehead and the drinking glass. Kinematic data was filtered with 6-Hz second-order Butterworth filter in forward and backward direction and analyzed off-line in the Matlab software (R2019B, The Mathworks Inc).

The drinking task was divided into 5 phases: (1) reaching to grasp the glass, (2) forward transport of the glass to the mouth, (3) drinking a sip of water, (4) transporting the glass back on the table, and (5) returning the hand back to the starting position.

For the standardization of the sitting position, the chair and table height were adjusted to attain 90° knee and hip flexion, 90° elbow flexion while the upper arm was in vertical and forearm in horizontal position [17]. The wrist was aligned with the table edge with the palm resting on the table. A hard-plastic drinking glass containing 100 ml water was placed 30 cm from the table edge (approximately 75–80% of the arm’s length) in the midline of the body. The trunk was not restrained, although the participants were instructed to sit with their back against the back of the chair. After few familiarization trials, ensuring that the participants had understood the instructions correctly, the drinking task, including all 5 phases, was repeated in self-paced natural speed at least 10 times unimanually, starting with the dominant or less-affected arm. The rest between each trial was approximately 5 s.

A set of kinematic variables describing both temporal and spatial characteristics of the movement performance, including end-point, angular and displacement variables, were obtained for the analysis. Definitions of the kinematic variables are provided in Table 2.

**Table 2:** Definitions of the kinematic variables of the drinking task

Kinematic variables	Definitions
*Temporal and end-point kinematics*	
Total movement time (MT) (s)	Time is calculated for the entire drinking task and separately for each phase. The start and end of the movement was defined as the point in time when the velocity exceeded or was below 2% of the maximum velocity in the reaching or returning phase, respectively. Detailed definitions for each phase are available in a previous publication [17]
MT reaching (s)	
MT forward transport (s)	
MT drinking (s)	
MT back transport (s)	
MT returning (s)	
Number of movement units total (NMU)	Movement units were computed from the tangential velocity profile separately for first two movement phases (reaching and forward transport), last two phases (back transport and returning) and as a summed total of these four phases (NMU total). One movement unit was defined as a difference between a local minimum and next maximum that exceeded the amplitude limit of 20 mm/s, minimum time between two subsequent peaks was set to 150 ms. NMU indicates movement smoothness
NMU phase 1&2	
NMU phase 4&5	
Peak velocity (mm/s)	Peak tangential velocity of the hand marker in the reaching phase
Time to peak velocity (%)	Percentage of time to peak velocity in the reaching phase; indicates relative time spent in acceleration and deceleration
Peak elbow angular velocity (°/s)	Peak angular velocity in the elbow joint (extension) in the reaching phase
*Angular and displacement kinematics (arm and trunk)*	
Shoulder abduction reaching (°)	Maximum shoulder angle in frontal and sagittal plane, between the vectors joining the shoulder and elbow markers, and the vertical vector from the shoulder marker toward the hip
Shoulder abduction drinking (°)	
Shoulder flexion reaching (°)	
Shoulder flexion drinking (°)	
Elbow extension reaching (°)	Minimum or maximum angle of the elbow joint between the vectors joining the elbow and wrist markers, and the elbow and shoulder markers
Elbow flexion drinking (°)	
Wrist angle (°)	Maximum angle of the wrist joint in reaching and forward transport phase between the vectors joining the hand and wrist marker, and the wrist and elbow marker
Inter-joint coordination, r	Temporal cross-correlation between the shoulder flexion and elbow extension during the reaching phase. Stronger correlation indicates that joint motions are coupled
Trunk displacement (mm)	Maximum displacement of the thorax marker from the initial position during the entire drinking task

*MT* movement time, *NMU* number of movement units

### Statistical analysis

The software Matlab (Mathworks Inc, R2018b) was used for all statistical analyses. Kinematic data from 10 trials was available for 68% and 78% of the non-disabled and stroke participants, respectively. All remaining sessions had 9 successful trials. Hence, in the analysis of performance stability, systematic trends and test–retest reliability 9 trials were used. Three trials from two non-disabled participants showed distinctively lower values of the inter-joint coordination. A visual analysis confirmed that these deviating values were caused by a backward movement of the hand prior forward reaching and these trials were therefore excluded from analysis.

The performance stability was verified through analysis of reliability, i.e. the repeatability of the selected kinematic variables. The intraclass correlation coefficient (ICC) was used to assess this. The ICC was calculated from the ratio between variance of interest and the total variance which gives a value between 0 and 1, where 1 represents excellent reliability. The ICC can be computed in different ways depending on which variance that is analyzed [29]. In this case we were interested in the stability of the average measure from a set of repetitions. The ICC analyzing absolute agreement for average measurements in a sample of random individuals [29] was selected and used to determine the number of trials needed to reach performance stability for each variable. The ICC values were calculated separately for the non-disabled participants and participants with stroke, but also for data from the two groups together. For the latter combined ICC scores, the non-dominant arms of the non-disabled participants and more-affected arms of the individuals with stroke were used.

Thresholds for the ICC were set according to recommendations by Koo and Li [29], which are based on the 95% confident interval of the ICC estimate. Values of ICC were interpreted as poor (less than 0.50), moderate (0.50- < 0.75), good (0.75–0.90), and excellent (greater than 0.90).

In order to determine the number of trials needed to reach good reliability, a series of ICC was calculated for each variable, where each ICC in the series represents the ICC value based on n consecutive trials (n = 1,…,9). The ICC that reached ≥ 0.75 gave the recommended number of trials for each variable.

The systematic within-session trend was investigated by comparing the average of trial 1–3 with the average of trial 7–9 from the same occasion. A paired t-test was used, and the significance level.

p ≤ 0.05 was used to reject the null hypothesis that no trend existed. To control for multiple comparisons, p values were adjusted with Holm’s correction [30].

The test–retest reliability of kinematic variables was analyzed in a subset of five persons with chronic stroke who had repeated the drinking task at four occasions with one week between each occasion. The measurements in persons with stroke were obtained during an assessment phase prior an intervention and were considered as stable. The test–retest reliability was analyzed by computing an individual average for each person, variable and occasion based on n trials (n = 1,…,9). The ICC that represented the absolute agreement for single measurements was used (since the average computed for each occasion was defined as a single measure) to determine the number of trials needed to reach good test–retest reliability for each variable in this subgroup. The same threshold levels were used as when analyzing performance stability, i.e. ICC ≥ 0.75 represented good test–retest reliability.

## Results

Background characteristics of the participants are shown in Table 1. There were no statistically significant differences between the non-disabled participants and individuals with stroke in terms of age, sex, body height and weight. All participants were right hand dominant.

### Performance stability

**Fig. 1:**
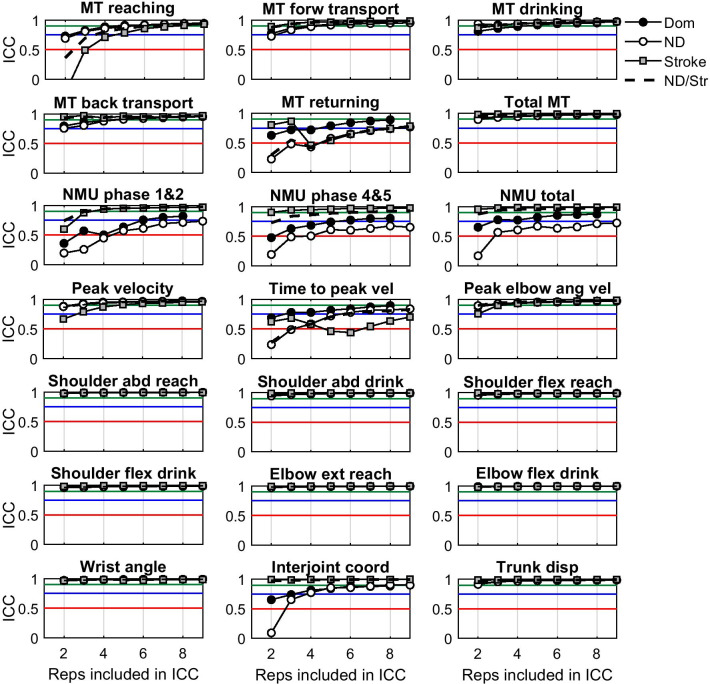
The ICC representing performance stability are plotted as a function of the number of included repetitions. The horizontal lines represent poor (0.5, red), good (0.75, blue) and excellent reliability (0.90, green), respectively. *MT* movement time, *NMU* number of movement units, *vel* velocity, *ang* angular, *abd* abduction, *flex* flexion, *ext* extension, *coord* coordination, *disp* displacement, *reps* repetitions, *Dom* dominant arm in non-disabled group, *ND* non-dominant arm in non-disabled group, *ND/Str* data from non-dominant arm in non-disabled and more affected arm in stroke combined

The values for all kinematic variables for dominant and non-dominant arms in non-disabled and for the more affected arm in persons with stroke are reported in Table 3. ICC values as a function of number of included trials needed to reach good performance stability of kinematic measures are shown in Fig. 1. Number of trials needed to reach good performance stability are summarized in Table 4. The combined ICCs (non-dominant arms of the non-disabled participants and more-affected arms of the individuals with stroke) revealed that 18 of 21 variables reached good to excellent reliability for averages based only on 2 to 3 trials. More trials were needed for Movement time (MT) reaching (4 trials), MT returning (8 trials) and Time to peak velocity (6 trials). In the analyses of the non-disabled group alone the results were similar except for Number of Movement Units (NMU, 3 to > 9 trials) and Inter-joint coordination (4 trials). Even when only 3 trials were needed for NMU total of the dominant arm to reach good reliability, 9 or more trials were required for NMU of the non-dominant arm. The between-individual variations for these variables were low in the non-disabled group compared to the participants with stroke (see standard deviations reported in Table 3). In the separate analysis with participants with stroke alone, more than 3 trials were needed for MT reaching (5 trials), MT returning (8 trials) and Time to peak velocity (> 9 trials).

**Table 3:** Group means (SD) for the kinematic variables

Kinematic variables	Non-disabled (n = 44)		Stroke (n = 8)
	Dominant arm	Non-dominant arm	More affected arm
*Temporal and end-point kinematics*			
Total movement time (MT) (s)	6.0 (0.9)	6.2 (0.9)	8.2 (2.2)
MT reaching (s)	0.99 (0.17)	0.98 (0.17)	1.19 (0.39)
MT forward transport (s)	1.2 (0.2)	1.2 (0.2)	1.9 (0.8)
MT drinking (s)	1.3 (0.3)	1.4 (0.4)	1.7 (0.6)
MT back transport (s)	1.5 (0.2)	1.6 (0.3)	2.3 (1.0)
MT returning (s)	1.1 (0.2)	1.1 (0.2)	1.2 (0.2)
Number of movement units total (NMU)	5.7 (0.9)	6.2 (0.7)	10.8 (4.5)
NMU phase 1&2	2.2 (0.3)	2.3 (0.3)	4.7 (2.5)
NMU phase 4&5	3.5 (0.7)	4.0 (0.6)	6.1 (2.3)
Peak velocity (mm/s)	651 (116)	632 (91)	560 (85)
Time to peak velocity (%)	41 (6)	42 (8)	42 (5)
Peak elbow angular velocity (°/s)	99 (23)	105 (22)	68 (26)
*Angular and displacement kinematics (arm and trunk)*			
Shoulder abduction reaching (°)	24.2 (6.5)	22.0 (6.2)	26.3 (7.0)
Shoulder abduction drinking (°)	32.1 (10.6)	28.6 (9.7)	37.7 (14.7)
Shoulder flexion reaching (°)	43.4 (6.0)	43.2 (5.8)	41.7 (8.6)
Shoulder flexion drinking (°)	49.8 (6.5)	49.9 (5.8)	52.3 (9.0)
Elbow extension reaching (°)	55 (7)	54 (8)	63 (14)
Elbow flexion drinking (°)	135 (5)	135 (5)	132 (5)
Wrist angle (°)	28 (6)	30 (6)	30 (5)
Inter-joint coordination, r	− 0.96 (0.05)	− 0.97 (0.04)	− 0.73 (0.62)
Trunk displacement (mm)	33 (15)	35 (17)	70 (58)

*MT* movement time, *NMU* number of movement units

**Table 4:** Number of trials needed to reach good performance stability and test–retest reliability (ICC > 0.75)

	Performance stability (within-session)				Test–retest
	Non-disabled (n = 44)		Stroke (n = 8)	All (n = 52)	Stroke (n = 5)
	Dominant arm	Non-dominant arm	More-affected arm	Tested arm*	More-affected arm
*Temporal and end-point kinematics*					
Total movement time (s)	**2**	**2**	**2**	**2**	**2**
MT reaching (s)	**3**	**3**	5	4	**3**
MT forward transport (s)	**2**	**3**	**2**	**2**	**2**
MT drinking (s)	**2**	**2**	**2**	**2**	**2**
MT back transport (s)	**2**	**2**	**2**	**2**	**2**
MT returning (s)	5	8	8	8	–
NMU total	**3**	–	**2**	**2**	**2**
NMU phase 1&2	6	9	**3**	**2**	**2**
NMU phase 4&5	6	–	**2**	**3**	**2**
Peak velocity (mm/s)	**2**	**2**	**3**	**2**	–
Time to peak velocity (%)	**3**	6	–	6	–
Peak elbow angular velocity (°/s)	**2**	**2**	**2**	**2**	**2**
*Angular and displacement kinematics (arm and trunk)*					
Shoulder abduction reaching (°)	**2**	**2**	**2**	**2**	**2**
Shoulder abduction drinking (°)	**2**	**2**	**2**	**2**	**2**
Shoulder flexion reaching (°)	**2**	**2**	**2**	**2**	**2**
Shoulder flexion drinking (°)	**2**	**2**	**2**	**2**	**2**
Elbow extension reaching (°)	**2**	**2**	**2**	**2**	**2**
Elbow flexion drinking (°)	**2**	**2**	**2**	**2**	**2**
Wrist angle (°)	**2**	**2**	**2**	**2**	6
Inter-joint coordination, r	4	4	**2**	**2**	**2**
Trunk displacement (mm)	**2**	**2**	**2**	**2**	**2**

Values in bold indicate good performance stability and test–retest reliability; no value indicates that more than 9 trials were needed

*MT* movement time, *NMU* number of movement units

^*^Included non-dominant arm in non-disabled and more affected arm in stroke

### Systematic trend over a set of trials

**Fig. 2:**
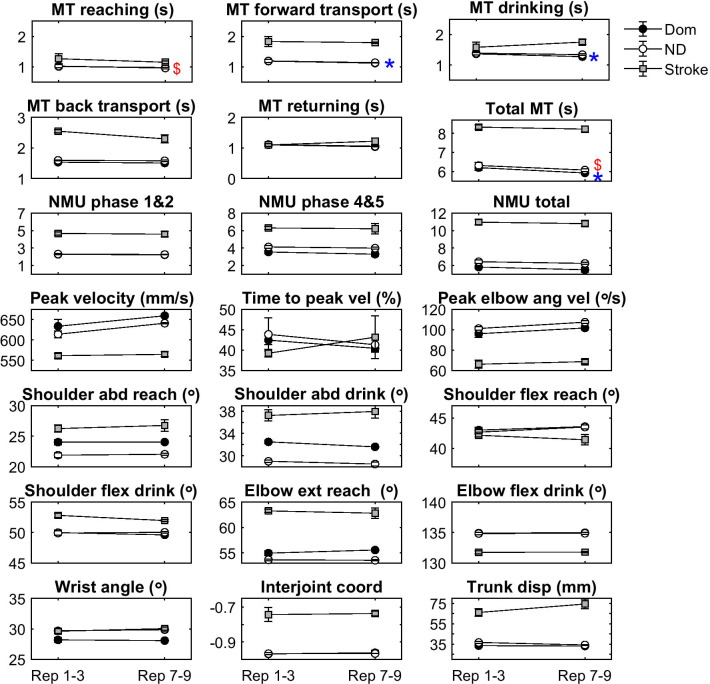
Trends in terms of number of trials needed for good performance stability over a set of trials in the kinematic variables of the drinking task. *Indicates statistical difference in dominant and $ in non-dominant arm in the group of non-disabled participants. No statistical differences were found for the stroke group. *MT* movement time, *NMU* number of movement units, *vel* velocity, *ang* angular, *abd* abduction, *flex* flexion, *ext* extension, *coord* coordination, *disp* displacement, *reps* repetitions, *Dom* dominant arm in non-disabled group, *ND* non-dominant arm in non-disabled group

The systematic within-session trends between the first 3 trials (trial 1–3) and the last 3 trials (trial 7–9) are shown in Fig. 2. Small but significant trends (p < 0.001) were observed in movement time variables in the non-disabled group, while no trends were found in the stroke group.

### Test–retest reliability in a subgroup of individuals with stroke

**Fig. 3:**
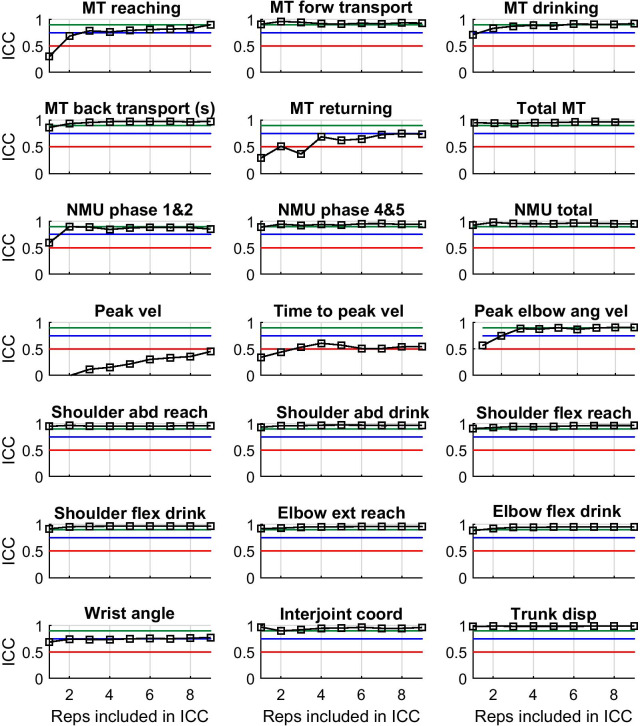
Test–retest reliability expressed as Intra-class correlation (ICC) calculated for the number of trials included in the average value from each of four occasions (n = 20) in a subgroup of five persons with stroke. The horizontal lines represent poor (0.5, red), good (0.75, blue) and excellent test–retest reliability (0.90, green), respectively. *MT* movement time, *NMU* number of movement units, *vel* velocity, *ang* angular, *abd* abduction, *flex* flexion, *ext* extension, *coord* coordination, *disp* displacement, *reps* repetitions

In the subset of five participants with hemiparesis after stroke, 17 out of 21 variables showed good or excellent test–retest reliability if the average value from each occasion were computed from 2 to 3 trials (Fig. 3 and Table 4). For MT returning > 9 trials were needed. For the Wrist angle variable, the ICC was close to 0.70 after 2 trials, but reached over the level of ≥ 0.75 after 6 trials. The reliability remained moderate for Time to peak velocity over the 9 trials and for Peak velocity the reliability remained poor (Fig. 3).

## Discussion

This study determined the minimum number of trials needed to reach good performance stability of kinematic variables obtained during the drinking task both in non-disabled persons and in a sample of individuals with chronic stroke. The results revealed that for most kinematic variables only 2 to 3 trials were required to reach sufficient performance stability. Small but significant trends were noted for shorter movement times in the non-disabled group for the last 3 trials compared to the first 3 trials. In the stroke sample, a good to excellent test–retest reliability was reached for many variables when less than 3 trials from each occasion were used in the analysis. However, more trials were needed for movement time in reaching and returning as well as for wrist angle. Only moderate reliability was reached for the time to peak velocity and poor reliability was observed for the variable peak velocity in the stroke group.

### Number of trials needed to reach good performance stability

The current study is the first to demonstrate that only 2 to 3 trials are required to reach good performance stability for most kinematic variables of the drinking task. This finding was valid both for non-disabled and for stroke participants and is in line with two previous studies analyzing pointing and reaching kinematics using optoelectronic systems [21, 22]. Blinch et al. reported that not more than 3 trials were required to achieve good within trial reliability of movement time and peak velocity during fast visually guided pointing tasks in non-disabled participants [21]. Likewise, Hansen et al. demonstrated that 5 trials were estimated to be the minimum number required to get reliable ICC estimates for most of the kinematics when reaching for low and high targets in persons with subacute stroke [22].

Similar results have also been shown with other measurement systems in non-disabled individuals. A study using a virtual reality gaming Kinect system showed that 2 to 5 trials during reaching were needed to achieve performance stability in movement time and elbow and shoulder range of motion [31]. Additionally, when using an inertial sensor system, comparable results of 3 trials was considered enough to reach acceptable levels of reliability for movement time and shoulder and elbow range of motion during a drinking task in non-disabled participants [32]. These results confirm that for most of the kinematic variables a set of 3 trials would be sufficient. However, more trials in a range of 4–6 and ≥ 8 trials would probably be needed for certain variables and study groups (e.g. non-disabled participants).

 Even though the total movement time for the drinking task only required 2 trials to reach good performance stability, up to 5 trials were needed for movement time in reaching (stroke) and up to 8 trials for movement time during returning (stroke and non-disabled). Post-stroke, abnormal muscle activation synergies and inadequate inter-joint coordination have been suggested to be the prime contributing causes to reaching dysfunction [10, 33, 34]. In addition, abnormal inter-segmental dynamics, particularly regarding suppressed interaction torque and deficient feedforward control of this torque around the elbow might significantly contribute to the dysfunction in reaching [35]. Deficits in the grasp formation during reaching impact as well the reaching time [36]. All these complex demands on reaching might increase the within trial variability in reaching seen in individuals with stroke.

To move the hand back to the starting position in the returning phase of the drinking task should theoretically be less challenging, however, up to 8 trials were needed to reach good performance stability in both investigated groups. One possible explanation for this finding could be that the movements in this phase did not require direct visual feedback and that the participants might have corrected the end position of the hand in some trials. To overcome this potential problem, a more standardized end of the task could be used.

The relative time to peak velocity, designating acceleration and deceleration time in reaching, showed also higher variability with 6 or more trials required to reach good performance stability in both groups. Higher variability, characterized by lower effect sizes of discriminative validity, was also observed for this variable during the drinking task in persons with stroke in a previous study [17]. This suggests that this point in time when the peak velocity is reached may vary between trials both in persons with stroke and in those without disability.

Interestingly, in the non-disabled group more trials were needed for NMU (3 to 9 and more) and inter-joint coordination (4 trials) than in individuals with stroke (2–3 trials). The main reason for that was most likely the inherent properties of the variables themselves. In both metrics, the between-subjects’ variation was extremely low compared to participants with stroke (see Table 3). Further, the performance of non-disabled participants was also close to the extreme possible value of the metrics (ceiling or floor effect). These aspects need to be considered when interpreting the reported ICC values for these variables in the non-disabled group.

Good movement performance stability was reached after 2 trials for all joint angles and trunk displacement metrics (Fig. 1 and Table 4). This finding confirms that movement variability of the joints and segments of the body is relatively stable when repeatedly performing a well-known task [16], such as drinking from a glass, in a self-paced comfortable speed. This result is in line with previous research in non-disabled persons showing high level of automaticity of movement execution of well-learned tasks [16], and also in persons late after stroke where compensatory movement strategies have shown to be more fixed [37, 38].

### Systematic trend over a set of trials

In the non-disabled individuals, small but significant trends towards improvement were demonstrated in some temporal variables (for total movement time and for some of the movement phases) when the last three trials were compared to the first three. These trends might be caused by the learning effect. The improvements were, however, small and can therefore be considered to be of less clinical relevance.

In the stroke group, no significant trends over multiple trials were found, but even here small trends could be observed visually in some variables, e.g. increased trunk displacement in later trials (Fig. 2). Not finding significant trends in stroke data could be caused by the low power due to the small group size (n = 8), and larger studies in stroke populations are therefore warranted.

We expected to find signs of muscular fatigue in terms of declining trends in the stroke group over the set of trials, but this assumption was not supported in the results. Interestingly, from an intervention study it was reported that participants in post stroke training could conduct up to 300 repetitions (3 tasks × 100 reps)/occasion, within one hour) without experiencing increased fatigue [39]. The risk of fatigue influencing motor performance after stroke has, however, been highlighted in several previous studies [12, 20, 22], and a planned rest in between trials has been recommended. In the current study, the participants took a short break of about 5 s between each trial.

### Test–retest reliability in a subsample of individuals with stroke

In the current study, good to excellent test–retest reliability with a mean of 2 to 3 trials was demonstrated for most of the kinematic variables in the individuals with stroke performing the drinking task at 4 different occasions. However, for two end-point variables (the peak velocity and the time to peak velocity), the reliability remained poor or moderate even after 9 trials. Our findings agree with previous research [19, 20], even though there are some methodological differences.

In a study with participants with stroke (tested at two occasions, few days apart) good to excellent test–retest reliability were found for movement time, peak velocity and trunk displacement in different reach-to-grasp tasks (different object sizes and at self-selected and fast speeds) [19]. Interestingly, for non-disabled controls only moderate to good reliability was demonstrated [19]. The authors proposed that the lower consistency observed in non-disabled individuals might be caused by an exploratory behavior among controls trying to find the most optimal solutions for movement execution within the existing task constraints [40]. Individuals with hemiparesis after stroke often move with behavioral compensation and this altered movement performance has been reported to be less variable [11, 37, 41]. From a theoretical dynamic system’s perspective, the underlying mechanisms for these more fixed movement patterns developed over time in people with stroke might explain the low observed variations [38].

Test–retest reliability of kinematic variables obtained during a pointing task, using a mean of 2 trials in persons late after stroke, showed varying ICC values [20]. Good reliability (ICC > 0.75) was reported for shoulder flexion and elbow extension, moderate reliability for peak velocity, shoulder abduction and inter-joint coordination, while the ICC values for movement time, time to peak velocity and number of velocity peaks were low [20]. In contrast to the Wagner et al. [20], our results showed good reliability for movement time (except for the returning phase) and NMU, while the time to peak velocity showed low reliability similarly to the abovementioned study. Plausible explanations to these inconsistent results might be the differences in tasks and that the participants in the Wagner et al. study had more impaired upper extremity function (FMA ≈ 35/66) as compared to in the current study (FMA ≈ 50/66). The time between test–retest sessions was also longer (one month) in the study of Wagner et al. compared to one week in the current study, which might have influenced the results.

### Strengths and limitations

In the current study a wide range of well-established kinematic variables covering temporal, end-point, angular and displacement kinematics were evaluated, which is a strength of the study. The results regarding non-disabled people were based on a relatively large sample (n = 44), although the results from stroke participants need to be interpreted with caution due to the small sample size (n = 8) and particularly regarding the results of test–retest reliability where data from 4 test occasions in 5 participants was available. Nevertheless, kinematic data was available from repeated test occasions, giving 23 and 20 kinematic data sets available for analysis of within-trial reliability and test–retest reliability, respectively. The results in stroke participants should, however, be used as first evidence and future studies with larger sample size in stroke are needed to confirm our results. In this context, the findings from the current study suggest that 3–5 trials per test occasion can be used as a guide for self-paced functional everyday reach-to-grasp tasks both in non-disabled people and in individuals with stroke.

As also experienced in the current study, not all trials might be successful during the data capture due to various reasons including obscured markers and data gaps. This might be particularly relevant for individuals with stroke where the altered movement patterns might cause obscured markers resulting in data gaps. This further suggests that even when a good performance stability might be reached with 2 to 3 trials, few extra trials are needed to ensure sufficient number of successful trials.

The results of the current study are only applicable for the kinematic motion capture systems using multiple optoelectronic cameras. The results seem, however, to be similar even when the kinematics are collected by other systems, such as Kinect camera or inertial sensors [31, 32]. This is promising, taking the constant push from users (clinicians, researchers and patients) to make movement analysis more readily available with systems that can operate outside the lab.

## Conclusions

This is the first study that determines the number of trials needed to achieve good performance stability and test–retest reliability for multiple kinematic variables during a drinking task in persons with and without upper extremity impairments. The findings demonstrated that only 2–3 trials were needed for most of the kinematic variables to reach good within-session performance stability, both in non-disabled and in a sample of individuals with chronic stroke. Good to excellent test–retest reliability (comparing 4 occasions) was reached in a subgroup of individuals with stroke. These results imply that a recommendation for future studies to collect at least 3 trials of each tested condition is well founded and applicable for most of the kinematics. However, there are few exceptions, and in these cases a larger number of trials is warranted. The results are primarily applicable for the drinking task, but partly also to other similar purposeful reach-to-grasp tasks.

## Data Availability

The datasets used and/or analyzed during the current study are available from the corresponding author on reasonable request.
